# A variance component analysis on recombination rate in the COGA pedigrees

**DOI:** 10.1186/1471-2156-6-S1-S58

**Published:** 2005-12-30

**Authors:** Lin Wang, Xin Xu

**Affiliations:** 1 Population for Population Genetics, Harvard School of Public Health, Boston, MA 02115, USA

## Abstract

We estimated the crossover frequency in 1,232 gametes from 356 subjects in pedigrees from the Collaborative Study on the Genetics on Alcoholism. We examined the effect of covariates including age, ethnicity, and years with ALDX1 on recombination rate, and found a positive correlation between recombination rate and years with ALDX1. By variance-component analysis, we estimated the heritability of recombination rate to be around 0.5, and provided suggestive evidence for a locus linked to recombination rate.

## Background

Crossover between homologous chromosomes in meiosis is the most important mechanism for shuffling the genetic material in humans. It has been estimated that the average number of crossover events per gamete is 26.5 in males and 39 in females, respectively [1]. While variation of recombination rate along chromosomes has been characterized quite extensively [2-4], few studies have explored the environmental and genetic factors explaining the inter-individual variation in recombination rate. This is partially due to the technical difficulties in accurately counting the number of crossovers in large samples. However, the number of crossover in each gamete can be estimated in pedigrees with genotype data on high-density markers across the genome. In this report, we analyzed the pedigree genotype data from the Collaborative Study on the Genetics on Alcoholism (COGA) that was made available for the Genetic Analysis Workshop 14 (GAW14) with the goals to 1) estimate the effects of ethnicity, age, and alcohol drinking on meiotic recombination rate; 2) estimate the heritability of recombination rate; and 3) perform linkage analysis on recombination rate.

## Methods

### Study subjects and genotyping

Details of the GOGA data has been described elsewhere. In brief, it consists of 143 pedigrees of variable size and different ethnic origin. Besides gender, age, and ethnicity, phenotypic data includes a selected set of phenotypes related to alcohol dependence. We selected the DSM-III-R+Feighner classification status for alcohol dependence (ALDX1) and onset age of ALDX1 for this study.

The COGA pedigrees have been genotyped using three screening sets: the conventional microsatellite markers, the Illumina SNP screening set, and the Affymetrix 10K single-nucleotide polymorphism (SNP) set. Genotypes of 11,120 SNPs from the cleaned Affymetrix 10K SNP set, available in 1,316 COGA subjects, were used in our analysis due to their high density of SNPs.

### Counting of meiotic crossover frequency

We first phased the genotypes of each pedigree members using GENEHUNTER [5]. We then detected the occurrence of crossover in the gamete of each parent by comparing the parental and offspring haplotypes. We only evaluated those gametes with observed genotypes in both the individual and his/her offspring. Double recombinations (DRs) within a 1-cM interval were discarded because they are most likely due to genotyping error. The so-called 'double recombinants' include two recombination events in one parent as well as one recombination event in each of the two parents. A total of 15,429 DRs out of 54,041 scored were deleted. The female-to-male ratio of such double recombinants was 1.09 while the ratio of non-DR events was 1.69. This is consistent with the general belief that most of these DRs were the result of genotype errors that have no sex preference. For easier computation, we split large chromosomes into consecutive 250-SNP blocks and split large pedigrees (>22 bits) into smaller ones.

### Statistical analysis

We estimated the effects of covariates including gender, age, ethnicity, and alcohol dependence on individual recombination rate with a multiple linear regression model using R 1.8.1. Generalized estimating equation (implemented in R package "gee") were used to adjust for correlations due to observations of multiple gametes from the same individual. The individual recombination rate was calculated as the total number of observed crossovers in 22 autosomes. The variable "age" was defined as the difference of the ages at interview between the individual and his/her offspring, which should approximate the age at gamete production. We let variable "dage" be the difference between age (at gamete production) and onset age of ALDX1 (if observed) in an individual. The years with alcohol dependence at gamete production age ("adyr") was defined as equal to 0 if ALDX1 = 1 ("pure" unaffected) or dage ≤ 0 or identical to dage if ALDX1 = 5 (affected) and dage > 0. If neither criteria held, adyr was recorded as missing.

We carried out variance-component analysis on the recombination rate using GENEHUNTER v2.1, estimated the heritability of recombination rate, and performed linkage analysis under the presumption of no dominant polygenic variance [6]. Before the variance-component analysis, we normalized individual recombination rate by gender-specific means and variances. We also repeated the analysis using MERLIN, which produced a very similar result.

## Results

**Figure 1:**
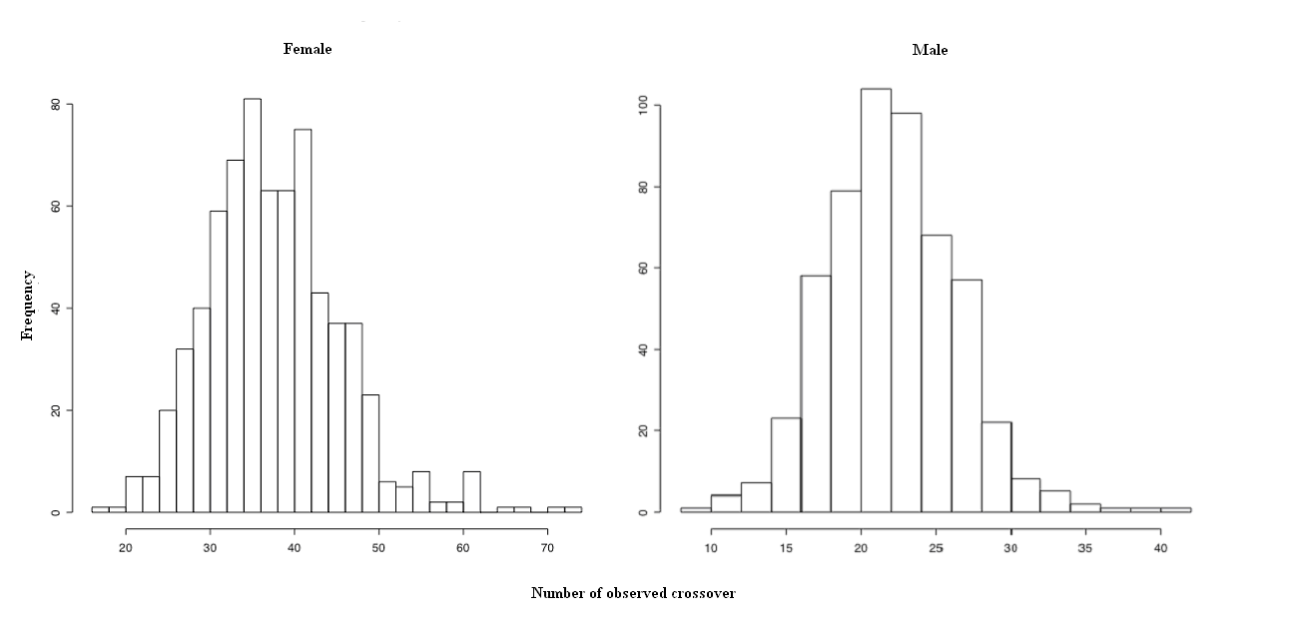
Distribution of observed gender-specific overall crossover frequency in 22 autosomes.

With the Affymetrix 10K SNP genotypes in COGA pedigree, we were able to estimate crossover frequency in a total of 1,232 gametes from 356 individuals, including 34 non-Hispanic Blacks, 286 non-Hispanic Whites, and 21 Hispanic Whites. The average of male, female, and sex-averaged crossover rate was 22.6, 38.1, and 31.1, respectively. The sex-specific distribution is shown in Figure 1. Age (at gamete production) was moderately correlated with adyr (*r*^2 ^= 0.24). The effects of covariates age, gender, ethnicity, and adyr on recombination rate, estimated by multiple linear regression, is summarized in Table 1. Besides the prominent gender effect, recombination rate increased over the years with ALDX1 at gamete production at a rate of 1 crossover per gametes per 20-year of ALDX1 (*p *< 0.05). Both age at gamete production and ethnicity did not have a significant effect on recombination rate. Gender-stratified analysis produced very similar estimates of the gender-specific coefficients for age and adyr.

The heritability of recombination rate estimated from variance component analysis was 51.3%. Of 143 COGA pedigrees, only 38 pedigrees had one or more sibling pairs or half-sibling pairs with observed recombination rate. Our linkage analysis identified 9 loci with LOD > 1 (Table 2). The highest peak was located on chromosome 16 with a LOD score of 2.1.

## Discussion

Our analysis on recombination rate in 1,232 gametes from 356 subjects in the COGA study suggests that recombination rate increases over the years with ALDX1 (adyr). We further provided evidence, for the first time, for a substantial genetic component in recombination rate, and identified a few candidate loci for recombination rate.

Though statistically significant, the effect size of adyr on recombination rate is quite small. It is not clear if this effect is the direct consequence of alcohol dependence or an indirect result from alcohol drinking for which adyr serves as a surrogate marker. It would be very interesting to examine the effect of alcohol drinking, but such data at the age of gamete production was not available.

Our estimates of number of crossovers per gamete in both sexes, and particularly in males, are a little lower than those from other studies. Several possible explanations for the slight under-estimation of recombination rate are: 1) the genetic markers in this study may not cover the entire autosome; 2) despite the high density of Affymetrix 10K SNPs, many SNPs with small or no heterozygosity provide little information on genotype phasing, resulting in omission of a small portion of double recombination between informative markers; and 3) the rule to discard double recombination within a 1-cM interval may lead to discarding a few true double reanalyze them.

The major weakness of our variance component linkage analysis is lack of power due to insufficient sample size. Recombination rate can only be determined for individuals who have genotyped offspring. As a result, only 38 out of 143 COGA pedigrees are informative for this linkage analysis. It would be very desirable to pool all available large genome scan studies with 3-generation pedigrees and reanalyze them.

## Conclusion

Through our study, we estimated that the heritability of recombination rate was 51.3% using variance component analysis. We also provided suggestive evidence for a locus linked to recombination rate on chromosome 16.

## Abbreviations

COGA: Collaborative Study on the Genetics on Alcoholism

DR: Double recombination

GAW14: Genetic Analysis Workshop 14

SNP: Single-nucleotide polymorphism

## Authors' contributions

LW carried out the variance component analysis and regression analysis, and drafted the manuscript. XX conceived of the study, took charge in study design, and draft the manuscript. Both authors read and approved the final manuscript.

## Figures and Tables

**Table 1:** Coefficients in the regression model of recombination rate^a^

	Estimate	SE	Z	P
Intercept	38.48	1.35	28.34	0
Male	-15.67	0.59	-26.58	0
Black, non-Hispanic	1.66	1.016	1.64	0.101
White, Hispanic	-0.71	0.75	-0.95	0.342
Other ethnicity	-0.14	0.94	-0.14	0.889
Age	-0.025	0.046	-0.55	0.582
Adyr^b^	0.049	0.022	2.21	0.027

^a^White, non-Hispanic is the reference group for ethnicity.

^b^The years with alcohol dependence at gamete production age

**Table 2:** Result of variance component linkage analysis

		Variance		
			
Chr	Position (cM)	Additive QTL	Shared environmental factors	LOD
1	259.84	0.55	0.37	1.04
2	22.43	0.65	0.30	1.09
3	40.55	0.57	0.36	1.23
6	4.00	0.61	0.33	1.25
9	47.00	0.70	0.25	1.66
11	65.21	0.67	0.28	1.31
16	83.32	0.80	0.18	2.06
21	24.48	0.59	0.35	1.14
